# Inter-rater reliability of three standardized functional tests in patients with low back pain

**DOI:** 10.1186/1471-2474-10-58

**Published:** 2009-06-02

**Authors:** Johan Tidstrand, Eva Horneij

**Affiliations:** 1 Department of Health Sciences, Division of Physiotherapy, Lund University, Lund, Sweden; 2 Läkargården Physiotherapy Clinic, Midgårdsgatan 1, 262 71 Ängelholm, Sweden

## Abstract

**Background:**

Of all patients with low back pain, 85% are diagnosed as "non-specific lumbar pain". Lumbar instability has been described as one specific diagnosis which several authors have described as delayed muscular responses, impaired postural control as well as impaired muscular coordination among these patients. This has mostly been measured and evaluated in a laboratory setting. There are few standardized and evaluated functional tests, examining functional muscular coordination which are also applicable in the non-laboratory setting. In ordinary clinical work, tests of functional muscular coordination should be easy to apply. The aim of this present study was to therefore standardize and examine the inter-rater reliability of three functional tests of muscular functional coordination of the lumbar spine in patients with low back pain.

**Methods:**

Nineteen consecutive individuals, ten men and nine women were included. (Mean age 42 years, SD ± 12 yrs). Two independent examiners assessed three tests: "single limb stance", "sitting on a Bobath ball with one leg lifted" and "unilateral pelvic lift" on the same occasion. The standardization procedure took altered positions of the spine or pelvis and compensatory movements of the free extremities into account. The inter-rater reliability was analyzed by Cohen's kappa coefficient (κ) and by percentage agreement.

**Results:**

The inter-rater reliability for the right and the left leg respectively was: for the single limb stance very good (κ: 0.88–1.0), for sitting on a Bobath ball good (κ: 0.79) and very good (κ: 0.88) and for the unilateral pelvic lift: good (κ: 0.61) and moderate (κ: 0.47).

**Conclusion:**

The present study showed good to very good inter-rater reliability for two standardized tests, that is, the single-limb stance and sitting on a Bobath-ball with one leg lifted. Inter-rater reliability for the unilateral pelvic lift test was moderate to good. Validation of the tests in their ability to evaluate lumbar stability is required.

## Background

Only 15% of patients with low back pain (LBP) are given a specific pathological diagnosis, the remaining 85% are diagnosed as "non-specific lumbar pain" [1]. For this latter group, treatment as well as the evaluation of treatment are considered difficult. Postural control as well as muscular coordination of the lumbar spine, are found to be impaired among patients with lumbar pain [2-5]. Muscular reactions are delayed among these patients [6-8]. This has mostly been measured and evaluated in a laboratory setting [2-4,6-8]. Traditionally, lumbar instability has been defined merely as an osteological, mechanical instability verified by x-ray, for example spondylolisthesis [9]. However, in line with a more functional and broader definition of lumbar stability, researchers have pointed out the importance of muscular and neurological functions as well as those of the passive structures such as the vertebrae, cartilage and ligaments [10-15]. Panjabi describes the "neutral zone" as "a region of intervertebral motion around the neutral posture where little resistance is offered by the passive spinal column". The neutral zone has beed found to increase with injury and degeneration and decrease with muscle force [10]. An important aspect of good spinal stability function is to keep the spine in the neutral zone [10]. Bergmark devided the muscles, engaged in stabilizing the lumbar spine, into two separate systems, that is, the local system and the global system [13]. To keep the spine stabile during functional movements there has to be a coordinated interaction between the two systems [12,13]. In this study, each individuals capacity for keeping the spine in the neutral position was defined as "functional muscular coordination".

Clinicians use a battery of different clinical tests to investigate muscular coordination of the lumbar spine [16-19]. These tests must be reliable and valid. Clinical tests such as pain provocation tests [19], segmental mobility tests and test of functional muscular coordination [5,17-21] have been reported in the literature to classify patients with a lumbar instability diagnosis. In primary care, patients with non-specific low back pain, with or without associated radiating pain, are frequently encountered. Empirically, many of these patients have an impaired function of the proximal lumbar muscles. To our knowledge, there are few standardized and evaluated functional tests examining functional muscular coordination of the lumbar spine which can be used clinically. The aim of the present study was to therefore standardize and examine the inter-rater reliability of three functional tests of muscular functional coordination of the lumbar spine in patients with low back pain (LBP).

## Methods

### Subjects

Patients consulting a physiotherapist due to low back pain with or without referred pain in the lower limb, or for arm and/or shoulder pain were recruited from a private physiotherapy clinic in the south of Sweden. Inclusion criteria consisted of age group between 18–65 years and a good knowledge of the Swedish language. Exclusion criteria were pregnancy, visual analogue scale (VAS) > 70 mm, previous known trauma or operation of the lower extremity which could jeopardise the performance of the tests, and known neurological or rheumatological disease. Between each test the patients rated their pain on a VAS scale. The purpose of these ratings was to check that the score did not exceed 70 mm on the scale, which we considered too high and could potentially affect the performance of the next test.

The raters were blinded as to what diagonoses the patients had. Patients with low back or arm and/or shoulder pain were included. The purpose of including patients with shoulder pain was to avoid the raters being biased and also to obtain a more even distribution of positive/negative findings [22]. A list of the inclusion/exclusion criteria was given to the administrators or the physiotherapists at the setting, other than the raters concerned. Patients who fulfilled the inclusion criteria were consecutively offered participation in the study

The assessment undertaken in this study consisted of in part a normal orthopaedic objective assessment. The aims, methods and procedures of the study were approved by the head of the clinical setting. In accordance with Swedish law, the study complied with the ethical principles of the Helsinki Declaration. All patients gave their written, informed consent before participation. To decline participation in the study or to withdraw consent to participate did not affect patients' right to further rehabilitation. Statistical analyses were made on group level and no single individual could be identified.

### Procedure

The study was single-blinded, that is the raters were not informed of the patients' symptoms.

The patients were examined by two experienced physiotherapists (JT and AJ), both trained in orthopaedic manual therapy and in The McKenzie method [23]. Both physiotherapists had more than five years of experience of treating patients with lumbar instability.

Before each test, the patient was informed about the test procedure. This information was based on a standardized written protocol which was read by one of the physiotherapists. It was decided by randomization which physiotherapist should inform the patients about the performance of the tests. The same physiotherapist also demostrated how the tests should be performed. The randomization was done by computer. The tests were performed in the same order for all patients.

Before the study began, the physiotherapists co-coordinated the evaluation of the tests on ten patients in order to improve concordance. The results of these tests were not included in this study of reliability.

The inter-rater reliability was assessed for each test, at the same time, by the two physiotherapists. A study form was independently filled in by the physiotherapists. Before and after each test, the patients rated their pain from the low back, leg or arm/shoulder on a VAS scale. Before each test the physiotherapists asked the patients whether they scored >70 on the VAS scale, which was an exclusion criterion. After the tests had been completed the patients were examined and treated as in normal clinical practice.

### Tests

All the tests were started in the "neutral zone of the back" position, and therefore, the normal curve of the spine had to be identified [10,11]. In order to evaluate the curvature of the spine, the patients were inspected from behind and from the side independently by the two physiotherapists. The neutral position of the spine was defined as the starting position. To be sure the participants had understood the instructions of how to practice the tests they took up each test position once, before the assessment of each test. Each test was performed once on both sides right and left and each test position was maintained for 20 s. The two raters evaluated the tests at the same occasion. Every test began with the right leg against the floor or the couch, followed by left. Twenty seconds was considered to be enough time to evaluate the tests without causing too much inconvenience to the patient. According to clinical practice all tests were performed subsequently without any rest between.

#### Single-limb stance

**Figure 1:**
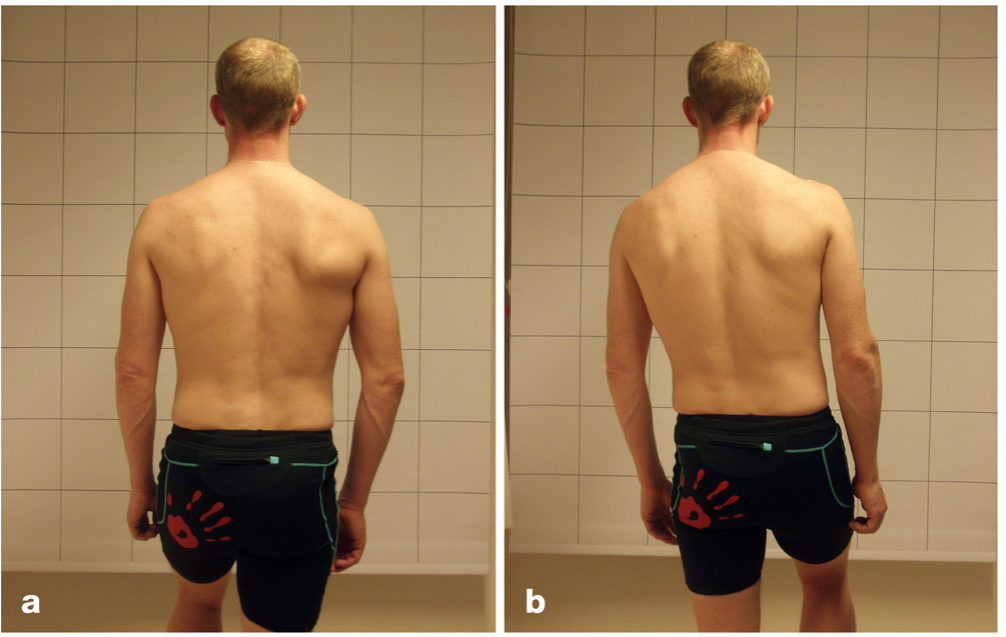
**Single-limb stance**. (a) Negative test result. (b) Positive test result.

Figures 1a and 1b show the single-limb stance position. The patient stood one metre in front of a checked curtain so that one of the longitudinal lines of the curtain was in line with the spine. The raters were positioned approximately two metres behind the patient as close to the patients midline as possible. The raters were kneeling down so that the eyes of the raters were in a horizontal line with the patient's low back. The contra-lateral leg was lifted about 60° of flexion at the hip. The patient was asked to stand with the spine as vertical as possible and with the arms hanging down. The test was evaluated as:

*Negative *if the spine was kept in its original vertical position for 20 s, and the pelvic crests were kept in their original horizontal plane for 20 s with no compensatory movements made by the contra-lateral leg or the arms. A change from the starting position was accepted as long as this position was quickly resumed.

*Positive *if:

• the spine deviated from the original vertical position and/or

• the pelvic crests deviated from the horizontal plane and/or

• compensatory movements were made by the contra-lateral leg or the arms and/or

• two or more short changes from the starting position were made.

*Not valid *if the patient did not manage to perform the test due to pain

#### Sitting on a "Bobath Ball" (large gymnastics ball)

**Figure 2:**
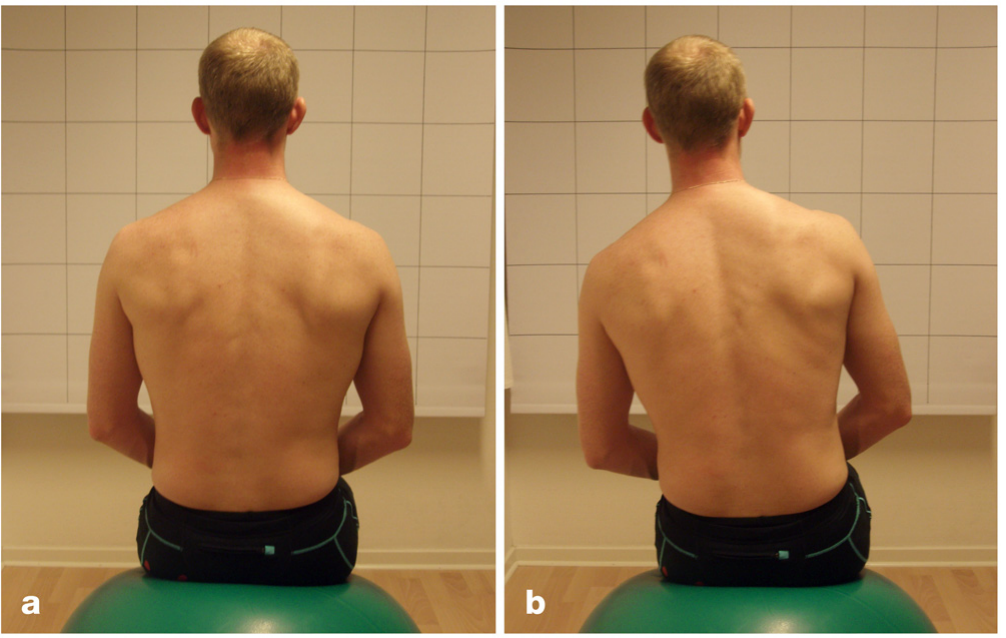
**Sitting on a Bobath ball**. (a) Negative test result. (b) Positive test result.

The patient sat on a Bobath ball one metre in front of a checked curtain so that one of the longitudinal lines of the curtain was in line with the spine. The raters were positioned about two metres behind the patient as close to the patients midline as possible. The raters were kneeling down so that the eyes of the raters were in a horizontal line with the patient's low back. The diameter of the ball was 0.65 m. However, for patients shorter than 1.60 m a ball 0.55 m in diameter was used and for patients taller than 1.90 m the diameter of the ball was 0.75 m. The dorsal sides of the patient's hands were loosely placed on his/her thighs. There was approximately 0.05 m between the feet, and the calves did not touch the ball. The patient was then asked to lift his/her foot and keep it about 0.05 m above the floor for 20 s (Figure 2a and 2b). The test was evaluated as:

*Negative *if the spine was kept in its original vertical position for 20 s and if no compensatory movements were made by the lifted leg or by the arms. A short change from the starting position was accepted as long as this position was quickly resumed.

*Positive *if:

• the spine deviated from the original vertical position and/or

• compensatory movements were made by the lifted leg or by the arms and/or

• two or more short changes from the starting position were made.

*Not valid *if the patient did not manage to perform the test due to pain.

#### Unilateral pelvic lift

**Figure 3:**
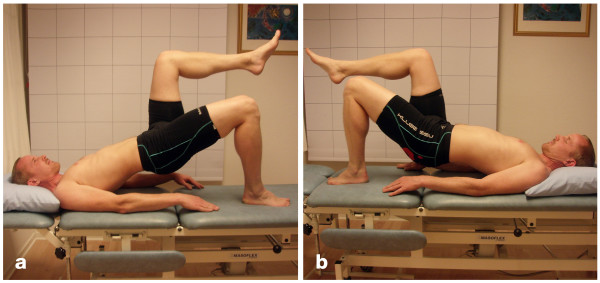
**Unilateral pelvic lift**. (a) Negative test result. (b) Positive test result.

Figures 3a and 3b show the unilateral pelvic lift. The raters were positioned about one metre beside the couch on the supported side. The raters were kneeling down so that the eys of the raters were in a horizontal line with the patient's. hip. The patient was in the supine position on a couch with the supporting leg flexed at the hip and the knee and arms resting parallel to the trunk on the couch. The contra-lateral leg was flexed to 90° at the hip and the knee. The patient was asked to press the supporting foot against the couch and lift the pelvis up so that the trunk was in line with the thigh of the supporting leg and so that an imagined line between both superior iliac spines of the pelvis was in the horizontal plane.

The test was evaluated as:

*Negative *if the imagined line between both superior iliac spines of the pelvis was in the horizontal plane for 20 s and the trunk was kept in line with the thigh of the supporting leg for 20 s. A short change from the starting position was accepted as long as this position was quickly resumed.

*Positive *if:

• the whole pelvis was lowered in the direction to the couch and/or

• the iliac spine of the pelvis on the side of the lifted leg deviated from the horizontal plane and/or

• compensatory movements were made by the lifted leg or by the arms and/or

• two or more short changes from the starting position were made.

*Not valid *if the patient did not manage to perform the test due to pain.

### Statistical analysis

The simplest way to study agreement between two raters is to calculate the percentage agreement or the absolute agreement. In order to account for the calculation of chance we used the kappa value [22]. As the kappa value depends on the prevalence of findings in each category, the ultimate situation is a 50% prevalence of positive findings [22,24]. As this not always is the case, the kappa value was complemented by the percentage agreement.

Each test was evaluated dichotomously: negative/positive. The inter-rater reliability was assessed by the percentage agreement and by Cohen's kappa [25]. The percentage agreement was calculated by dividing the numbers of agreed results with the total number of tests for each measured test. The kappa coefficient (κ) has a maximum of 1.0. A value of zero indicates agreement no better than chance. Negative values show less than chance agreement. According to the criteria of Altman [22], the kappa value was interpreted as follows: κ < 0.20 = poor, κ: 0.21–0.40 = fair, κ: 0.41–0.60 = moderate, κ: 0.61–0.80 = good, κ: 0.81–1.0 = very good. The analyses was carried out with SPSS for Windows (version 13.0).

## Results

Of all the nineteen patients (ten men) that were included, 13 had low back pain and six had arm and/or shoulder pain. Their mean age was 42 years (± 12 yrs). The patients with low back pain rated their pain on an ungraduated 100 mm visual analogue scale (VAS) where 0 = no pain and 100 = worst pain [26]. The mean value was 29 mm (± 17). Six of the patients were also suffering from pain radiating to the leg. The mean value of this pain was 35 mm (± 25).

Each patient carried out six tests, that is three for the left and three for the right side. Of all 114 tests that were evaluated only two were not valid due to pain. The results are presented in Table 1.

**Table 1:** Inter-rater reliability of three tests of muscular functional coordination of the lumbar spine.

**Single limb stance right**	AJ					
JT	Positive	Negative	Not valid	Totals	Percentage agreement	Kappa(κ)
Positive	2	0	0	2	100%	1.0
Negative	0	17	0	17		
Not valid	0	0	0	0		
Totals	2	17	0	19		

**Single limb stance left**	AJ					
JT	Positive	Negative	Not valid	Totals	Percentage agreement	Kappa (κ)
Positive	4	1	0	5	95%	0.88
Negative	0	13	0	13		
Not valid	0	0	1	1		
Totals	4	14	1	19		

**Sitting on a ball right**	AJ					
JT	Positive	Negative	Not valid	Totals	Percentage agreement	Kappa (κ)
Positive	9	2	0	11	89%	0.79
Negative	0	8	0	8		
Not valid	0	0	0	0		
Totals	9	10	0	19		

**Sitting on a ball left**	AJ					
JT	Positive	Negative	Not valid	Totals	Percentage agreement	Kappa (κ)
Positive	12	0	0	12	95%	0.88
Negative	1	6	0	7		
Not valid	0	0	0	0		
Totals	13	6	0	19		

**Unilateral pelvic lift right**	AJ					
JT	Positive	Negative	Not valid	Totals	Percentage agreement	Kappa (κ)
Positive	6	2	0	8	79%	0.61
Negative	2	8	0	10		
Not valid	0	0	1	1		
Totals	8	10	1	19		

**Unilateral pelvic lift left**	AJ					
JT	Positive	Negative	Not valid	Totals	Percentage agreement	Kappa (κ)
Positive	8	3	0	11	74%	0.47
Negative	2	6	0	8		
Not valid	0	0	0	0		
Totals	10	9	0	19		

Right/left means the supported side. Results achieved by rater AJ and rater JT. N = 19

According to the criteria of Altman [22], the inter-rater reliability for single limb stance was very good (κ: 1.0 and 0.88, respectively). Inter-rater reliability for the evaluation of the stability of the low back while sitting on a Bobath ball was good for the right leg (κ: 0.79) and very good for the left leg (κ: 0.88). The inter-rater reliability for unilateral pelvic lift was good for the right leg (κ: 0.61) and moderate for the left leg (κ: 0.47).

## Discussion

The present study showed that the single-limb stance and sitting on a Bobath ball with one leg lifted have a good to very good inter-rater reliability. The kappa value for unilateral pelvic lift was moderate to good. Despite the percentage agreement for this test (78% and 73% respectively) the raters disagreed on four and five tests out of 19 possible, which in our opinion is not an acceptable agreement. It might be possible to further adjust the standardization procedure for this test in order to improve to a more acceptable level.

As the kappa value depends on the prevalence of findings in each category, it is difficult to make a direct comparison to different studies of clinical tests. Murphy reports a kappavalue of 0.72 for left leg and 0.76 for right leg for a hip extension test [18]. Hicks reports a kappa value of 0.87 for Prone instability test, which is a pain provocation test proposed to identify patients with lumbar segmental instability [19]. Another aspect of lack of functional muscular coordination is an aberrant movement pattern during active trunk flexion [19]. For trunk range of motion the kappa value in this study was 0.69 for painful arc in flexion and 0.61 for painful arc on return of flexion. Other aspects of this dynamic movement showed bad agreement, that is Gower sign, instability catch and reversal of lumbopelvic rhythm [19].

The tests used in this study were measured from a neutral position of the spine in three different functional positions. They aimed at keeping the spine close to the neutral zone while maintaining the normal curvature of the spine through each test procedure [10,11]. An important aspect of the tests was the possibility to measure a difference between right and left side. The experience of the authors is that this difference has an important role in diagnosing/treating patients with a dysfunctional muscular coordination. The intention of the present study was to define and standardize three tests for functional muscular coordination. The standardization of the three tests was based on practical experience. The goal was to create simple tests which can be visually evaluated and which, beside a couch and a Bobath ball, can be performed without any special equipment. Whilst it may be considered a weakness, that we have not used objectively measurable quantities in our definitions of positive/negative test results the idea of our study was to show that it is possible to make visual standardizations which are reliable and allow physiotherapists to evaluate a change in the movement pattern of the low back. None of the tests, evaluated in the present study, have previously been described in this way. Thus, much effort was put into the standardization procedures, partly to create a distinct difference between negative and positive test results, and partly to distinguish a difference that is clinically applicable. A small change in the starting position was therefore accepted as negative as long as the starting position was quickly regained. However, if the patient remained in the changed position, the test was assessed as positive. Only two out of 114 tests were not valid because of pain, which illustrates the applicability of the tests. Interestingly, we noted a difference between the results for the left and the right side. Future studies could evaluate the validity of these tests and perhaps any correlation between differences of pain on the right and/or the left side.

Pain-ratings in this study were generally not affected by the tests. The patients did not rate their pain after the unilateral leg lifting test, which was the last one. We regretted this, as both raters found that of all the tests this one provoked the largest pain response. In this study, a checked curtain was used as reference in order to standardize the starting position. In a clinical setting, other backdrops could be used. Before the study started, the raters practiced their routine on ten patients, who were not included in the present study. They found that the evaluation of lumbar stability depended on where the rater was standing relative to the patient. In order to register a changed position, it was important to stand exactly behind the patient, which was a problem when two raters made the evaluation at the same time. When evaluating the unilateral pelvic lift, it was important to stand on the same side as the supported leg and to be in line (eye level) with the patient's hip to be able to evaluate any tipping of the pelvis and at the same time to have some reference line beneath the patient by which to register any lowering of the pelvis.

The Trendelenburg test was previously used to evaluate the strength of the abductor muscles of the hip [5,20,27]. The standardization of this test, proposed by Hardcastle and Nade [27], was also applied by Roussel et al. [20]. They evaluated the test for 30 s, the un-supported leg was flexed 30° at the hip whilst the same side part of the pelvis was to be lifted above the horizontal line. This position will automatically result in a non-neutral position for the lumbar spine, which was not the aim of the present study. In the study by Millisdotter et al. [5], the Trendelenburg test was performed for 20 s and with the un-supported leg flexed 60° at the hip. The evaluation of the test in their study was in agreement with our study concerning whether the pelvis was in the horizontal plane or whether compensatory movements were made by the lifted leg. However, they did not evaluate any spinal deviation from the vertical line as was done in this study. In conclusion, other authors have focused mostly on the patient's ability to keep the pelvis in the horizontal plane, while in this study a lateral shifting of the lumbar spine was included in the evaluation. This, in our opinion will give a more precise picture of the functional muscular coordination of the lumbar spine.

Exercising on a Bobath ball is regarded as a progression in training programs to gain control of the spine [28], where the aim of the exercise is, through a moveable support, to increase the degree of demand in the stabilization training. In this study, the aim of the Bobath ball test was to create a similar standardization as for the single-limb stance test but with reduced weight-bearing force on the lower leg/hip and thus, more specifically testing the low back. The two tests, the single-limb stance and sitting on a Bobath ball are similar as regards keeping the spine vertical and the pelvis in the horizontal position. Kavcic et al. [29] examined different positions traditionally used in stability training of the lumbar spine, and related these to the compressing forces on segment L4–L5 and the muscular stabilization effect. They showed that the compressing forces were very low when sitting on a Bobath ball, which indicates that this position would probably not cause pain. They also showed that the muscular forces required for stabilization in this position were low. In our study, the patients also lifted one leg in order to create a shearing force lateral to the lumbar spine, which in turn increases the load on the stabilization muscles on the contra-lateral side. The results of this study indicate that the muscular stabilization capacity is often different between the left and the right side of the spine. There were more positive signs when testing on a Bobath ball in comparison with the single-limb stance test. This may suggest a higher degree of difficulty when undertaken this exercise.

With the unilateral pelvic lift, other functions are also evaluated. Reliability was poorer for this test which may have been due to the difficulty in making a precise evaluation of whether the pelvis was being lowered or not. This may depend on how the raters were standing relative to the patient. Many patients seemed to lack a coordinated low back with many quick turns of the pelvis which also jeopardised the evaluations. For some patients it was difficult to achieve a good starting position due to tight hip flexor muscles. These patients therefore started with the pelvis in a lower position. This test has earlier been used to evaluate muscular endurance [21]. Shellenberg et al. [21] showed that patients suffering from low back pain had significantly impaired muscular endurance and that the muscles in action were mainly the erector spinae and the hamstring muscles. The unilateral pelvic lift has also been tested for dynamic endurance and evaluated by the pattern of compensatory movements, where a visual assessment was used to determine the patient's ability to raise the pelvis straight without side movements [5]. This test is usually performed in this way in exercise programs and is possibly a better way when evaluating the unilateral pelvic lifting test. Many of the patients experienced pain when performing the unilateral pelvic lift. Kavcic et al. [29] showed that the compressing forces on the segment L4–L5 as well as the forces of the stabilization muscles were greater when the unilateral pelvic lift was performed compared with sitting on a Bobath ball. However, the significance of a test performed in a position which is not often used in everyday life, may be questioned.

A weakness of this study is that the sample size was small which may affect the precision of the kappa value. Another weakness is that the evaluated tests are not validated. A strength of the study is that the standardization procedure, as performed in this study, has shown good to very good reliability between two raters for two out of three tests. Much effort was put into the standardization to simplify the evaluation of functional muscular coordination of the lumbar spine.

## Conclusion

The present study showed good to very good inter-rater reliability for two standardized tests, i.e. the single-limb stance and sitting on a Bobath-ball with one leg lifted. Inter-rater reliability for the unilateral pelvic lift test was moderate to good. Validation of the tests in their ability to evaluate lumbar stability is required.

## Competing interests

The authors declare that they have no competing interests.

## Authors' contributions

JT participated in the design of the study, participated in collecting the data, performed the statistical analyses, and drafted the manuscript. EH participated in the design of the study, the statistical analyses and in the progress and revision of the manuscript. Both authors read and approved the final manuscript.

## Pre-publication history

The pre-publication history for this paper can be accessed here:


